# Measuring daily time experience: development and validation of the Seven-Dimensional Time Quality of Life Scale

**DOI:** 10.3389/fpsyt.2026.1785368

**Published:** 2026-04-10

**Authors:** Bowen Xue, Yuetan Liu, Xiaoying Jiang, Zhe Ni, Xiaohua Sun, Hong Luo

**Affiliations:** 1 Affiliated Mental Health Center & Hangzhou Seventh People’s Hospital, Zhejiang University School of Medicine, Hangzhou, Zhejiang, China; 2 Department of Psychology and Behavioral Sciences, Zhejiang University, Hangzhou, China; 3 Research Center for Mental Health and Humanities, Zhejiang University School of Medicine, Hangzhou, Zhejiang, China

**Keywords:** daily time experience, mental well-being, scale development, time quality, validation

## Abstract

**Background:**

Mental health assessment has traditionally relied on symptom-based measures of depression and anxiety. Although widely used, such measures may overlook individuals’ everyday experiences and lifestyle contexts that are closely related to psychological well-being. Grounded in the Seven-Dimensional Time Theory (SDTT), the present study aimed to develop and validate the Seven-Dimensional Time Quality of Life Scale (SDT-QoLS) to assess the overall quality of daily time experience.

**Methods:**

Using a convenience sampling approach, participants aged 12–65 years were recruited from a psychiatric outpatient setting. Scale items were generated through literature review, qualitative interviews, and expert evaluation. A total of 608 valid questionnaires were included in the analyses. The sample was randomly divided into two subsamples for exploratory factor analysis (EFA; n = 304) and confirmatory factor analysis (CFA; n = 304). Factor structure, internal consistency, and convergent validity were examined.

**Results:**

EFA supported a unidimensional structure of the SDT-QoLS, accounting for 58.90% of the common variance. CFA indicated an acceptable model fit. The scale demonstrated good internal consistency (α = 0.906). SDT-QoLS scores were positively associated with mental well-being and negatively associated with depression, anxiety, and general psychological distress.

**Conclusion:**

The SDT-QoLS provides a brief and psychometrically sound measure of subjective daily time quality. By focusing on everyday time experience rather than psychological symptoms, the scale may complement existing mental health assessments and support research and practice in mental health promotion.

## Introduction

Time is a finite resource shared equally by all individuals, yet it is experienced and organized in markedly different ways. Daily time use structures routines, regulates biological rhythms, and influences emotional and behavioral patterns across everyday life. In recent years, health behavior research has increasingly adopted a “24-hour activity cycle” perspective, emphasizing the interdependence of sleep, sedentary behavior, and physical activity within a fixed temporal framework (1). This integrative perspective highlights that psychological and physical health are influenced not only by individual behaviors, but also by how daily time is distributed and experienced as a whole.

Empirical studies have shown that even modest reallocations of daily activities may be associated with meaningful changes in emotional well-being. For example, increases in moderate-to-vigorous physical activity accompanied by reductions in sedentary time have been linked to more positive affect on the same day (2). Regular daily routines have also been associated with greater emotional stability and psychological resilience, particularly under conditions of stress and uncertainty (3, 4). Conversely, disruptions to daily rhythms, such as those observed during the COVID-19 pandemic, have been consistently associated with poorer mental health outcomes (5). Together, these findings underscore the importance of the structure and regularity of daily time use in psychological functioning.

However, time is not only an objective resource measured in hours and minutes; it is also a subjective psychological experience. Individuals differ substantially in their perception of time passage, sense of control over daily schedules, and the extent to which their time use is experienced as meaningful or burdensome (6, 7). Accumulating evidence indicates that such subjective time experiences are closely related to emotional regulation, cognitive functioning, and overall well-being (8). Concepts such as time affluence further highlight that perceived quality of time, rather than time quantity alone, is associated with happiness and mental health (9).

Against this background, the Seven-Dimensional Time Theory (SDTT) has been proposed as a multidimensional framework for understanding daily time experience and its relationship with mental health (10). Rather than focusing solely on objective time allocation or single behavioral indicators, SDTT conceptualizes daily time experience as comprising several functionally distinct yet dynamically interrelated dimensions, including sleep, physical activity, focused engagement, social connection, interaction with nature, present-moment awareness, and self-reflection. From this perspective, psychological well-being is embedded within a broader *time ecology* through which individuals regulate biological rhythms, engage with their environment, and construct personal meaning in everyday life.

Despite growing theoretical interest in subjective time experience, standardized measurement instruments grounded in a comprehensive and integrative theoretical framework remain limited. Existing tools tend to assess isolated behaviors or symptom outcomes, rather than the overall quality of everyday time experience as an integrated construct. To address this gap, the present study aimed to develop and validate the Seven-Dimensional Time Quality of Life Scale (SDT-QoLS), with SDTT serving as a general theoretical reference. Specifically, this study examined the factorial structure, internal consistency, and convergent validity of the SDT-QoLS in a psychiatric outpatient sample.

## Methods

### Participants

Participants were recruited between January 7 and January 8, 2026, from the outpatient department of a psychiatric hospital in Hangzhou, Zhejiang Province, China, using a convenience sampling approach. Eligible participants were aged between 12 and 65 years. For participants younger than 18 years, written informed consent was obtained from their legal guardians prior to participation.

Exclusion criteria were: (a) severe mental disorders or organic neurological diseases; (b) conduct disorder–related behavioral problems; (c) severe physical illness (e.g., cancer or severe disability); (d) inability to complete the questionnaire due to cognitive or language impairment; and (e) acute suicide risk at the time of assessment.

Participants were recruited from the outpatient department of a psychiatric hospital in Hangzhou, Zhejiang Province, China, which serves a broad patient population across East China. The hospital has approximately 700,000 outpatient visits annually, with 1,500 beds at the main campus. The estimated average daily outpatient volume is approximately 1,918 visits. Data collection occurred from January 7 to January 8, 2026, using a convenience sampling approach. Eligible participants were aged 12 to 65 years. For participants younger than 18 years, written informed consent was obtained from their legal guardians prior to participation. A total of 705 questionnaires were distributed, and 650 valid questionnaires were ultimately included in the analyses, representing approximately 16.9% of the estimated outpatient visits during the survey period. The sample was randomly divided into two equal subsamples, with 304 participants assigned to exploratory factor analysis (EFA) and 304 participants assigned to confirmatory factor analysis (CFA).

### Measures

#### Seven-Dimensional Time Quality of Life Scale

The Seven-Dimensional Time Quality of Life Scale (SDT-QoLS) was developed as a brief measure of the subjective quality of individuals’ daily time experience, with the SDTT serving as a general theoretical reference (10). The scale consists of seven items corresponding to core dimensions of daily time experience: sleep, physical activity, focused engagement, social connection, interaction with nature, present-moment awareness, and self-reflection. Participants rated the overall quality of their experience in each area during the past two weeks on a 10-point endpoint-anchored rating scale, in which only the two endpoints (1 and 10) were verbally labeled. Higher scores indicate better perceived time quality. A preliminary English translation is provided in **Supplementary Table S1** for reference.

#### Warwick–Edinburgh Mental Well-Being Scale

The Warwick–Edinburgh Mental Well-being Scale (WEMWBS; 11) is a 14-item self-report measure designed to assess positive mental well-being, including emotional functioning, psychological functioning, and interpersonal relationships. Items are rated on a 5-point Likert scale ranging from 1 (“none of the time”) to 5 (“all of the time”), with total scores ranging from 14 to 70. Higher scores indicate greater levels of positive mental well-being. In the present study, the WEMWBS demonstrated excellent internal consistency (α = 0.943).

#### General Health Questionnaire–12

The General Health Questionnaire–12 (GHQ-12; 12) was used to assess general psychological distress and overall mental health status. The scale consists of 12 items measuring symptoms related to anxiety, depression, social dysfunction, and loss of confidence. Items are rated on a 4-point Likert scale reflecting the severity of recent psychological difficulties. Total scores range from 0 to 36, with higher scores indicating greater levels of psychological distress. In the present study, internal consistency was good (α = 0.854).

#### Patient Health Questionnaire–9

The Patient Health Questionnaire–9 (PHQ-9) is a nine-item self-report scale assessing the frequency of depressive symptoms during the past two weeks (13). Each item is rated on a 4-point Likert scale ranging from 0 (not at all) to 3 (nearly every day), reflecting symptom frequency. Total scores range from 0 to 27, with higher scores indicating greater depressive symptom severity. In the present study, the PHQ-9 demonstrated excellent internal consistency (α = 0.905).

#### Generalized Anxiety Disorder–7

The Generalized Anxiety Disorder–7 (GAD-7) is a seven-item self-report measure assessing anxiety symptoms over the past two weeks (14). Each item is rated on a 4-point Likert scale ranging from 0 (not at all) to 3 (nearly every day), reflecting the frequency of symptom occurrence. Total scores range from 0 to 21, with higher scores indicating greater anxiety symptom severity. In the present study, the GAD-7 demonstrated excellent internal consistency (α = 0.921).

### Procedure

Data were collected using a secure web-based survey system developed and maintained by the affiliated medical institution. The platform is hosted on institutional servers and supports encrypted data transmission (HTTPS protocol) and restricted backend access, ensuring data security and confidentiality throughout the study process. Access to raw data was limited to authorized research personnel only. Participants were approached in the outpatient waiting area by trained research assistants and were informed about the voluntary nature of the study. Those who expressed interest were provided with a secure web link and completed the questionnaire using a designated device provided on site. No identifying information (e.g., name, phone number, or ID number) was collected. Before accessing the questionnaire, participants were presented with an electronic information page outlining the study purpose, procedures, potential risks and benefits, confidentiality protections, and the right to withdraw at any time without consequences. To enhance data quality, the system restricted submissions to one per IP address and prevented missing responses through forced-response settings, while allowing participants to discontinue at any time. Participants were instructed to complete the questionnaire independently without assistance.

### Data analysis

Data analyses were conducted using SPSS version 26.0 and AMOS version 24.0. Descriptive statistics were calculated for demographic characteristics and scale scores. Internal consistency reliability was assessed using Cronbach’s alpha coefficients for the total scale. EFA was performed on one randomly selected subsample to examine the underlying factor structure of the SDT-QoLS. Principal axis factoring was used as the extraction method, with Promax rotation applied given the expected correlations among factors. Sampling adequacy was evaluated using the Kaiser–Meyer–Olkin(KMO) measure and Bartlett’s test of sphericity. Factor retention was guided by eigenvalues greater than 1.0 and inspection of the scree plot. Factor loadings ≥ 0.40 were considered acceptable. CFA was conducted on the second subsample using maximum likelihood estimation to evaluate the fit of the hypothesized factor structure. Model fit was assessed using χ²/df, CFI, TLI, RMSEA, and SRMR. Acceptable model fit was indicated by χ²/df =3.00˜5.00, CFI and TLI ≥ 0.90, RMSEA ≤ 0.08. Convergent validity was examined by calculating Pearson correlation coefficients between SDT-QoLS scores and scores on the WEMWBS, GHQ-12, PHQ-9, and GAD-7. Correlation coefficients greater than 0.300 were considered indicative of acceptable convergent validity, consistent with conventional benchmarks in psychological research.

### Ethics approval and consent to participate

This study received ethical approval from the School of Public Health and Nursing of Hangzhou Normal University on November 6, 2025 (Ethical Review Certificate No. 2025080), and from the Ethics Committee of the Affiliated Mental Health Center & Hangzhou Seventh People’s Hospital, Zhejiang University School of Medicine on January 7, 2026 (Approval No. 2026-002). All procedures were carried out in accordance with the Declaration of Helsinki. Electronic informed consent was obtained from all participants prior to participation. For participants younger than 18 years, written informed consent was additionally obtained from their legal guardians. All data were collected anonymously and treated with strict confidentiality.

## Results

### Participants’ characteristics

The final analytic sample consisted of 608 participants, including 201 males (33.1%) and 407 females (66.9%). Participants ranged in age from 12 to 65 years, with a mean age of 22.14 ± 8.62 years. Of the total sample, 291 (48.0%) were adolescents and 317 (52.0%) were adults. In the adolescent group, 93 (31.9%) were male and 198 (68.1%) were female, with a mean age of 15.48 ± 1.71 years. In the adult group, 108 (34.0%) were male and 209 (66.0%) were female, with a mean age of 28.25 ± 7.86 years.

### Descriptive statistics of SDT-QoLS items

Descriptive statistics of the SDT-QoLS items and total scores for the adolescent group, adult group, and the total sample are presented in **Table 1**. Across the total sample, the mean total score was 31.35 ± 13.97.

**Table 1:** Descriptive statistics for SDT-QoLS items in adolescents, adults, and the total sample.

Item	Adolescents (n = 291), M ± SD	Adults (n = 317), M ± SD	Total sample (n = 608), M ± SD
A1	4.56 ± 2.19	4.78 ± 2.47	4.68 ± 2.34
A2	3.98 ± 2.23	4.26 ± 2.39	4.12 ± 2.32
A3	3.74 ± 2.21	4.17 ± 2.52	3.96 ± 2.38
A4	4.46 ± 2.50	4.96 ± 2.71	4.72 ± 2.62
A5	4.37 ± 2.40	5.00 ± 2.87	4.70 ± 2.67
A6	4.14 ± 2.37	4.79 ± 2.66	4.48 ± 2.54
A7	4.42 ± 2.46	4.93 ± 2.65	4.69 ± 2.57
SDT-QoLS	29.66 ± 12.73	32.89 ± 14.88	31.35 ± 13.97

### Item generation

Item development was guided by the SDTT (10). Relevant literature in health behavior, positive psychology, and mindfulness research was reviewed to inform the conceptual scope of the scale. In addition, qualitative data were collected through face-to-face interviews with 32 outpatient participants to capture lived experiences of daily time use. Participants were invited to reflect on how they organized and experienced their time across core domains of the SDTT, including sleep, physical activity, focused engagement, social connection, interaction with nature, present-moment awareness, and self-reflection. These narratives provided contextual grounding for item formulation and helped ensure alignment between theoretical constructs and experiential realities. Based on these themes and expert consultation, an initial item pool was generated and refined for clarity and conceptual relevance. The preliminary version of the SDT-QoLS consisted of seven items rated on a 10-point Likert scale assessing perceived time quality over the past two weeks.

### Content validity

Content validity was examined by a panel of six experts with doctoral-level training in psychology or related disciplines. Experts evaluated the relevance and representativeness of each item using a five-point Likert scale. The item-level content validity index (I-CVI) ranged from 0.833 to 1.00, and the scale-level content validity index (S-CVI) was 0.985, indicating high expert agreement and satisfactory content validity.

### Face validity test

Face validity was further assessed using a purposive sample of 10 outpatient participants to ensure clarity and comprehensibility at the user level. Participants reported that the items were clear, comprehensible, and consistent with their daily time experiences. No wording ambiguities or comprehension difficulties were identified, supporting the interpretability of the scale.

### Item analysis

Preliminary item analysis indicated satisfactory psychometric performance. Corrected item–total correlations ranged from 0.771 to 0.840 and were all statistically significant (p < 0.001), indicating good discrimination of individual items. Inter-item correlations ranged from 0.505 to 0.696, indicating acceptable internal consistency without excessive conceptual overlap. Detailed inter-item and item–total correlations are presented in **Table 2**.

**Table 2:** Inter-item and item–total correlations of the SDT-QoLS (N = 608).

Item	A1	A2	A3	A4	A5	A6	A7	Total
A1	1	0.677**	0.611**	0.505**	0.503**	0.550**	0.523**	0.771**
A2		1	0.696**	0.635**	0.557**	0.587**	0.594**	0.840**
A3			1	0.636**	0.580**	0.580**	0.600**	0.834**
A4				1	0.592**	0.544**	0.530**	0.796**
A5					1	0.575**	0.520**	0.778**
A6						1	0.651**	0.802**
A7							1	0.790**

**p < 0.001.

### Construct validity

EFA was conducted on a randomly selected subsample (n = 304) to examine the underlying dimensional structure of the SDT-QoLS. The KMO measure of sampling adequacy was 0.907, and Bartlett’s test of sphericity was significant, χ²(21) = 1226.546, p <.001, indicating that the data were appropriate for factor analysis.

**Figure 1:**
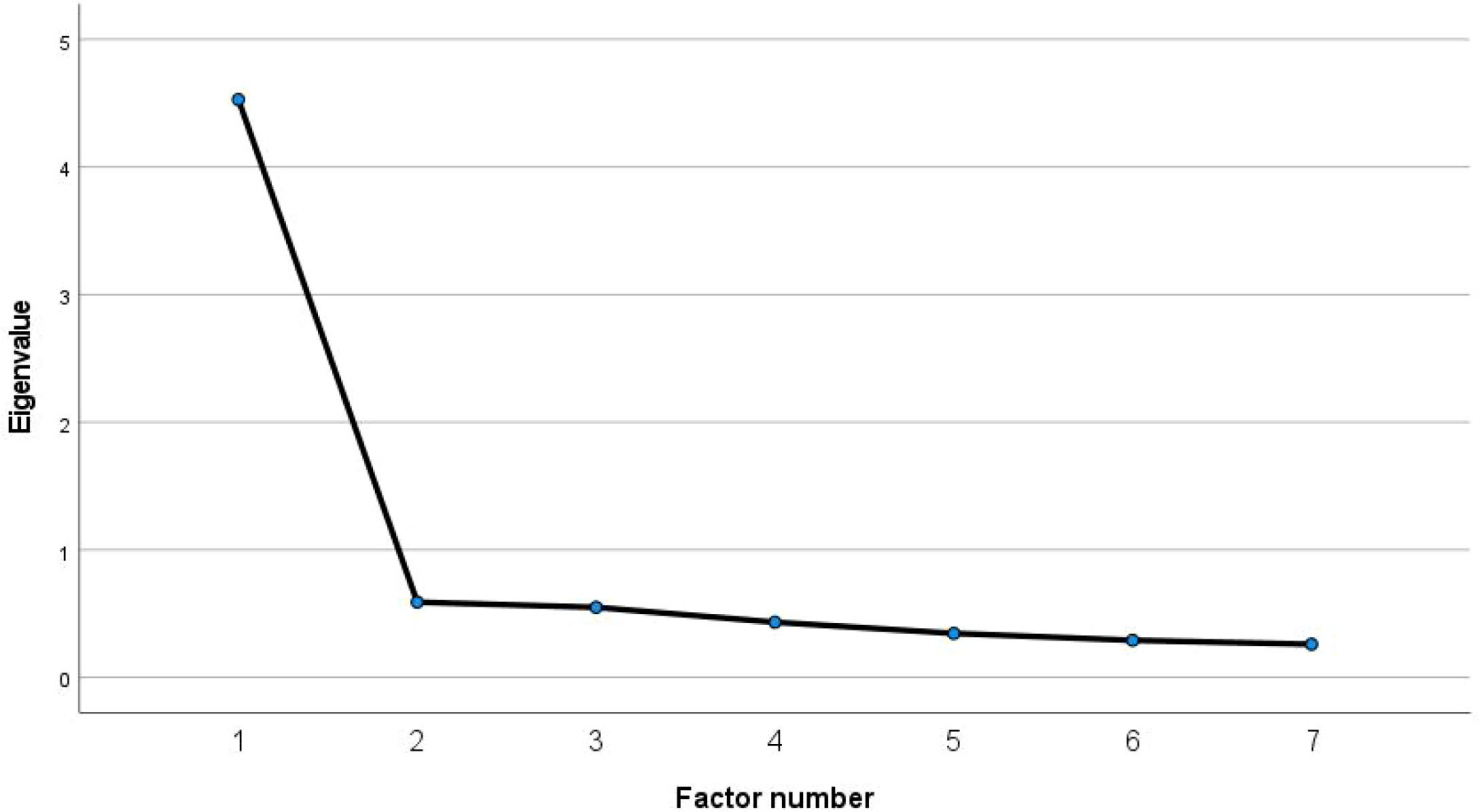
Scree plot of the SDT-QoLS.

Principal axis factoring supported a one-factor solution that accounted for 58.90% of the total variance. All items demonstrated substantial loadings on the single factor (0.719–0.838), with communalities ranging from 0.517 to 0.702. Collectively, these findings provide empirical support for a unidimensional structure of the SDT-QoLS. Detailed factor loadings and communalities are presented in **Table 3**, and the scree plot is shown in **Figure 1**.

**Table 3:** Factor loadings and communalities from exploratory factor analysis of the SDT-QoLS (n = 304).

Item	Communality (extraction)	Component 1 loading
A1	0.702	0.838
A2	0.635	0.797
A3	0.614	0.784
A4	0.575	0.758
A5	0.556	0.746
A6	0.523	0.723
A7	0.517	0.719

Extraction method: Principal Axis Factoring.

### Confirmatory factor analysis

CFA was conducted on the second subsample (n = 304) using maximum likelihood estimation to evaluate the hypothesized one-factor model. The model demonstrated an overall acceptable fit to the data (χ²/df = 3.232, CFI = 0.974, TLI = 0.960, NFI = 0.962, IFI = 0.974, RMSEA = 0.086).

**Figure 2:**
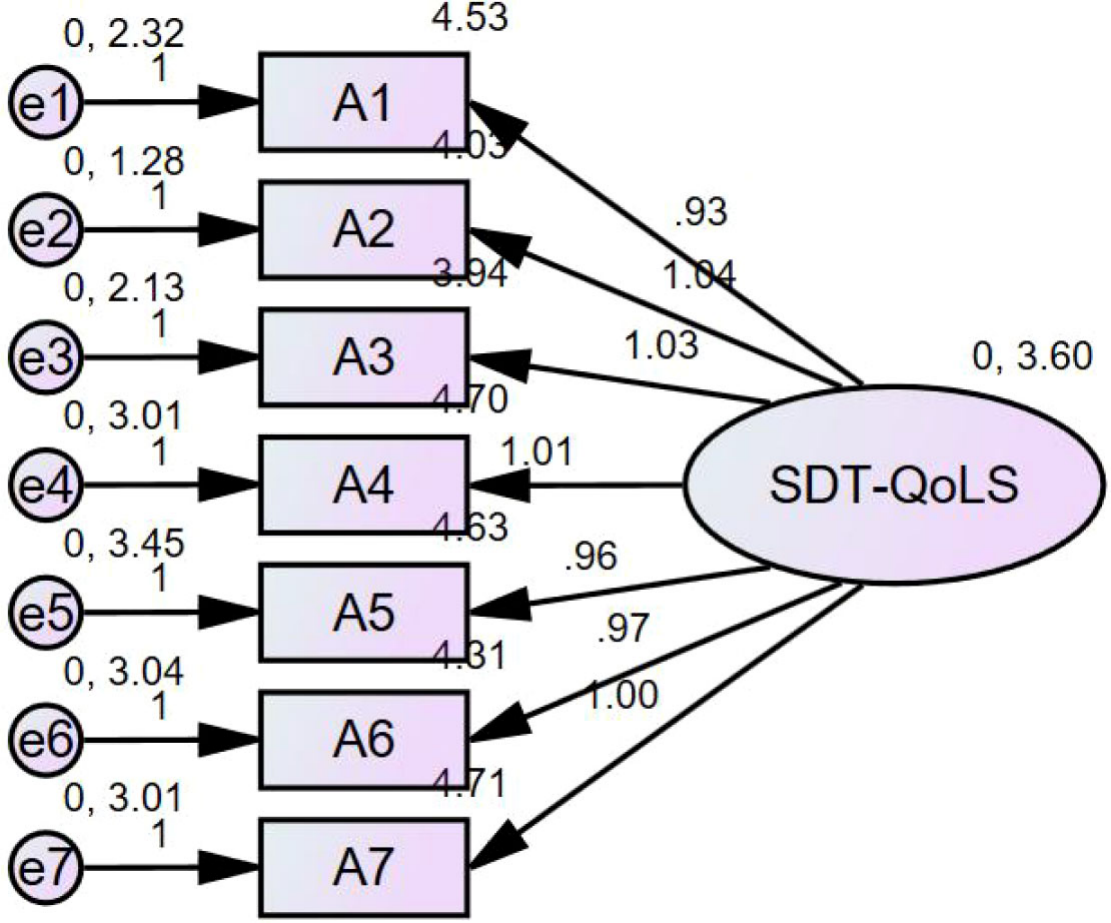
Scree plot of the SDT-QoLS.

Overall, these results support the structural validity of the SDT-QoLS. The standardized factor loadings for all items were statistically significant and ranged from moderate to high. The CFA model is illustrated in **Figure 2**.

### Reliability

The SDT-QoLS demonstrated good internal consistency reliability, with a Cronbach’s alpha coefficient of 0.906.

### Convergent validity

Convergent validity was first examined at the latent construct level using average variance extracted (AVE) and composite reliability (CR). The AVE was 0.583, exceeding the recommended threshold of 0.50, and the CR was 0.907, indicating adequate internal consistency and convergent validity at the factor level.

At the observed score level, Pearson correlation analyses were conducted to examine associations between SDT-QoLS scores and related constructs. As shown in **Table 4**, SDT-QoLS scores were strongly positively correlated with mental well-being (r = 0.824, p < 0.001) and strongly negatively correlated with general psychological distress (r = −0.707, p < 0.001), depressive symptoms (r = −0.781, p < 0.001), and anxiety symptoms (r = −0.710, p < 0.001).

**Table 4:** Correlations between the SDT-QoLS and measures of convergent validity.

Variables	SDT- QoLS	Well-being	GHQ-12	PHQ-9	GAD-7
SDT- QoLS	1				
Well-being	0.824**	1			
GHQ-12	-0.710**	-0.770**	1		
PHQ-9	-0.781**	-0.805**	0.792**	1	
GAD-7	-0.707**	-0.706**	0.732**	0.826**	1

All correlation coefficients are Pearson correlations. **p < 0.001.

### Age-stratified analyses

To further examine the structural robustness of the SDT-QoLS across developmental stages, exploratory and confirmatory factor analyses were conducted separately in adolescents (<18 years, n = 291) and adults (≥18 years, n = 317).

### Exploratory factor analysis

In the adolescent subgroup, principal axis factoring indicated sampling adequacy (KMO = 0.894; Bartlett’s test χ²(21) = 1022.035, p < 0.001) and supported a single-factor solution. The extracted factor accounted for 54.45% of the variance, with standardized loadings ranging from 0.682 to 0.830. Communalities ranged from 0.464 to 0.689 (see **Table 2**). In the adult subgroup, sampling adequacy was also satisfactory (KMO = 0.917; Bartlett’s test χ²(21) = 1368.799, p < 0.001). Principal axis factoring likewise supported a one-factor solution. The extracted factor accounted for 61.11% of the variance, with factor loadings ranging from 0.719 to 0.831 and communalities between 0.516 and 0.691 (see **Table 3**).

### Confirmatory factor analysis

Confirmatory factor analyses further supported the unidimensional structure in both subgroups. In adolescents, the model demonstrated acceptable fit (χ²/df = 4.930, CFI = 0.945, TLI = 0.891, NFI = 0.933, IFI = 0.946, RMSEA = 0.002). In adults, the model demonstrated good fit (χ²/df = 3.059, CFI = 0.980, TLI = 0.968, NFI = 0.971, IFI = 0.980, RMSEA = 0.081).

### Reliability

Internal consistency was good in adolescents (Cronbach’s α = 0.891) and excellent in adults (Cronbach’s α = 0.915).

### Convergent validity

Correlation patterns were highly consistent across developmental stages. In adolescents, SDT-QoLS scores were positively correlated with mental well-being (r = 0.799, p < 0.001) and negatively correlated with depressive symptoms (r = −0.777, p < 0.001), anxiety symptoms (r = −0.721, p <.001), and general psychological distress (r = −0.685, p < 0.001). In adults, SDT-QoLS scores were positively correlated with mental well-being (r = 0.777, p < 0.001) and negatively correlated with depressive symptoms (r = −0.781, p < 0.001), anxiety symptoms (r = −0.704, p < 0.001), and general psychological distress (r = −0.730, p < 0.001).

## Discussion

The present study developed and validated the SDT-QoLS based on the SDTT and examined its psychometric properties in a psychiatric outpatient sample. The findings provide consistent support for a unidimensional structure, satisfactory internal consistency, and convergent validity, suggesting that the SDT-QoLS functions as a reliable indicator of individuals’ perceived quality of daily time experience.

### Interpretation of the factor structure

Exploratory and confirmatory factor analyses consistently supported a single-factor structure underlying the SDT-QoLS. Although the scale items represent conceptually distinct domains of daily time experience (e.g., sleep, physical activity, focused engagement, and social connection), the unidimensional structure suggests that these domains cohere at the experiential level as an integrated construct of overall time quality. This finding aligns with the core assumption of the SDTT, which conceptualizes daily time experience as a holistic psychological resource rather than as a collection of independent behavioral components.

Importantly, the unidimensional structure does not imply that the individual domains are interchangeable or redundant. Instead, it indicates that, at the level of subjective evaluation, individuals may integrate multiple aspects of daily time experience into a global appraisal of how well their time is lived. From a measurement perspective, this structure supports the use of a brief composite score to capture overall time quality while preserving the possibility of more fine-grained dimensional assessments in future research.

### Associations with mental health indicators

The SDT-QoLS demonstrated strong positive associations with mental well-being and strong negative associations with depressive symptoms, anxiety symptoms, and general psychological distress. These findings indicate that higher perceived quality of daily time experience is closely related to better mental health status (15, 16). Notably, the magnitude of these correlations suggests that subjective time quality captures an aspect of everyday functioning that overlaps substantially with, yet remains conceptually distinct from, traditional symptom-based measures.

Unlike conventional mental health instruments that directly assess psychological symptoms, the SDT-QoLS focuses on individuals’ lived experiences within their daily routines and temporal organization. This difference in assessment focus may partly explain the strong associations observed, as everyday time experience constitutes a contextual layer through which psychological distress and well-being are expressed. At the same time, because the present study employed a cross-sectional design, these relationships should be interpreted as correlational rather than causal.

### Implications for mental health assessment and promotion

The findings of this study offer a complementary perspective for mental health assessment and promotion. First, the SDT-QoLS adopts a non-symptom-oriented approach, characterizing individuals’ mental health–related functioning through the lens of daily time experience. This perspective may hold practical relevance in contexts where symptom-based measures alone are insufficient to fully capture individuals’ everyday life functioning. By focusing on time-related domains such as sleep, physical activity, focused engagement, and social connection, the scale provides a holistic assessment framework that is closely aligned with lived daily experiences.

Second, the SDT-QoLS serves as a descriptive tool for examining associations between daily time structure and psychological states. In settings such as schools, community programs, or primary care contexts, the scale may be used to describe individuals’ overall patterns of perceived daily time quality, thereby offering contextual information that may inform supportive services or program development. Unlike traditional symptom-based instruments, the SDT-QoLS emphasizes subjective experiences of life rhythms and time allocation, contributing to a time-ecological perspective on psychological functioning.

In addition, future research may examine the seven conceptual dimensions within multidimensional frameworks. The present findings support a unidimensional structure at the level of global evaluation. The seven domains remain theoretically distinct and may show differential patterns across populations or contexts. Subsequent studies can test multidimensional models, bifactor models, or apply network analytic approaches to evaluate the relative contribution and interrelations of specific dimensions. Intervention-oriented research can investigate whether targeted changes in specific domains such as sleep regularity, physical activity, focused engagement, emotional awareness, or social connection are associated with changes in overall perceived time quality. Longitudinal or repeated-measures designs are needed to examine temporal dynamics and sensitivity to change across dimensions. Such research will refine the conceptual structure of time quality and clarify its scope of application in mental health research.

### Strengths and limitations

Several strengths of the present study merit attention. The SDT-QoLS was developed within a clearly articulated theoretical framework and demonstrated robust psychometric properties despite its concise format. The use of both exploratory and confirmatory factor analyses in independent subsamples strengthens confidence in the stability and replicability of the factor structure. Moreover, the strong associations observed with established measures of well-being and psychological distress provide further support for its convergent validity.

Nevertheless, several limitations should be acknowledged. First, the study employed a cross-sectional design, which precludes conclusions about causal relationships between time quality and mental health outcomes. Longitudinal studies are needed to examine the temporal dynamics and predictive validity of the SDT-QoLS. Second, participants were recruited from a single psychiatric outpatient clinic, and their daily time experience may be shaped by symptom burden, help-seeking processes, treatment engagement, and routine disruptions that are not representative of community or school-based populations. In addition, the present study did not report diagnostic subgroup distributions or examine differences across specific psychiatric diagnoses. Therefore, the psychometric evidence reported here supports the use of the SDT-QoLS primarily in similar clinical outpatient settings. Generalization to non-clinical populations or across diagnostic categories should be made with caution. Future studies should replicate the factor structure and validity in community and school samples, include multi-site recruitment across regions, and test measurement invariance across key demographic groups (e.g., age, sex) and clinical status. Such work will help clarify the scope and boundaries of the SDT-QoLS across different populations. Third, test–retest reliability was not examined in the present study. This was primarily due to the cross-sectional design and the clinical context of data collection, which limited the feasibility of follow-up assessments within a fixed retest interval. In addition, because the SDT-QoLS assesses perceived daily time quality over the past two weeks, short-term fluctuations in daily experiences may influence temporal stability estimates. Future studies employing longitudinal or repeated-measures designs are needed to examine the temporal stability of the SDT-QoLS and to determine its sensitivity to both natural changes and intervention-related effects. Finally, all data were collected using self-report measures, which may be subject to response biases.

## Conclusion

In conclusion, the present study provides initial empirical evidence that the SDT-QoLS is a brief, reliable, and theoretically grounded measure of subjective daily time quality. By shifting attention from psychological symptoms to everyday time experience, the scale offers a complementary perspective for mental health assessment. Future research is warranted to further examine its applicability across diverse populations and to evaluate its utility in longitudinal and intervention-based designs.

## Data Availability

The raw data supporting the conclusions of this article will be made available by the authors, without undue reservation.
