# Supplementary material for: Measuring daily time experience: development and validation of the Seven-Dimensional Time Quality of Life Scale
Source: Front Psychiatry. 2026 Apr 10;17:1785368. doi: 10.3389/fpsyt.2026.1785368 (PMC13106355; doi:10.3389/fpsyt.2026.1785368)
Supplement: Supplementary file 1 [file Table1.docx]

## Table S1 Seven-Dimensional Time Quality of Life Scale (SDT-QoLS)

(Preliminary English Translation – For Reference Only)

**Instruction:**
Please evaluate the quality of your daily time experience over the past two weeks. For each item, select a number from 1 to 10 that best reflects your experience. Only the two endpoints are verbally labeled: 1 indicates the lowest level of perceived time quality for that item, and 10 indicates the highest level. The response options from 2 to 9 represent intermediate levels between the two endpoints.

| **Item** | **Content** | **Response format** |
| --- | --- | --- |
| A1 | During the past two weeks, how would you rate your sleep quality and amount of sleep? Did you feel refreshed during the day?  这段时间的睡眠时长和质量如何？白天精力充沛吗？ | Extremely fatigued 1 — 2 — 3 — 4 — 5 — 6 — 7 — 8 — 9 — 10 Fully refreshed |
| A2 | During the past two weeks, did your body receive enough movement and stretching? Did you feel physically vital?  这段时间身体是否得到了充分的活动和舒展？是否感到活力流动？ | Sedentary and stagnant 1 — 2 — 3 — 4 — 5 — 6 — 7 — 8 — 9 — 10 Physically energized |
| A3 | During the past two weeks, did you experience periods of deep engagement or “flow” in work or study?  这段时间是否有全心投入、忘记时间的“心流”体验？工作效率或学习专注度如何？ | Distracted and unfocused 1 — 2 — 3 — 4 — 5 — 6 — 7 — 8 — 9 — 10 Fully concentrated |
| A4 | During the past two weeks, did you have opportunities to connect with sunlight, fresh air, plants, or animals? Did you feel connected to nature?  这段时间是否有机会接触阳光、新鲜空气、植物或动物？是否感受到了与自然的连接？ | Disconnected from nature 1 — 2 — 3 — 4 — 5 — 6 — 7 — 8 — 9 — 10 Deeply connected to nature |
| A5 | During the past two weeks, did you experience warm and sincere interpersonal interactions? Did you feel understood, supported, or able to care for others?  这段时间是否有一次温暖、真诚的交流？是否感到被理解、被支持，或给予了他人关爱？ | Lonely and emotionally distant 1 — 2 — 3 — 4 — 5 — 6 — 7 — 8 — 9 — 10 Emotionally fulfilled |
| A6 | During the past two weeks, were you aware of your emotions? When emotions arose, were you able to pause and observe them?  “这段时间你有没有注意自己的心情？当情绪来了，能不能先停一下，看一看它？” | Overwhelmed by emotions 1 — 2 — 3 — 4 — 5 — 6 — 7 — 8 — 9 — 10 Calmly aware of emotions |
| A7 | During the past two weeks, did you have quiet moments for reflection? Did you gain new insights about yourself?  “这段时间你有没有一些安静想一想的时刻？有没有在回想的时候，对自己有新的发现或体会？” | Mentally chaotic 1 — 2 — 3 — 4 — 5 — 6 — 7 — 8 — 9 — 10 Clear and insightful |

Note: The SDT-QoLS was originally developed and validated in Chinese. The English version provided here is a preliminary translation for reference only and has not undergone formal cross-cultural adaptation or psychometric validation. The officially validated version is the Chinese version. Permission from the corresponding author is required for reproduction, adaptation, or research use.

**Table S2. Exploratory Factor Analysis of the SDT-QoLS in Adolescents**(n = 291)

| Item | Communality | Factor Loading |
| --- | --- | --- |
| A2 | 0.689 | 0.83 |
| A3 | 0.641 | 0.801 |
| V6 | 0.529 | 0.727 |
| V7 | 0.506 | 0.711 |
| V5 | 0.499 | 0.706 |
| A1 | 0.484 | 0.696 |
| A4 | 0.464 | 0.682 |

**Extraction Method:** Principal Axis Factoring
**KMO:** 0.894
**Bartlett’s Test:** χ²(21) = 1022.04, p < .001
**Variance Explained:** 54.45%

## **Table S3. Exploratory Factor Analysis of the SDT-QoLS in Adults**(n = 317)

| Item | Communality | Factor Loading |
| --- | --- | --- |
| A2 | 0.691 | 0.831 |
| A3 | 0.685 | 0.828 |
| A4 | 0.633 | 0.795 |
| A6 | 0.597 | 0.773 |
| A7 | 0.579 | 0.761 |
| A1 | 0.577 | 0.759 |
| A5 | 0.516 | 0.719 |

Extraction Method: Principal Axis Factoring
KMO: 0.917
Bartlett’s Test: χ²(21) = 1368.799, p < 0.001
Variance Explained: 61.11%
